# The Long Journey of Pontine Nuclei Neurons: From Rhombic Lip to Cortico-Ponto-Cerebellar Circuitry

**DOI:** 10.3389/fncir.2017.00033

**Published:** 2017-05-17

**Authors:** Claudius F. Kratochwil, Upasana Maheshwari, Filippo M. Rijli

**Affiliations:** ^1^Chair in Zoology and Evolutionary Biology, Department of Biology, University of KonstanzKonstanz, Germany; ^2^Zukunftskolleg, University of KonstanzKonstanz, Germany; ^3^Friedrich Miescher Institute for Biomedical ResearchBasel, Switzerland; ^4^University of BaselBasel, Switzerland

**Keywords:** pontine gray nuclei, reticulotegmental nuclei, precerebellar system, cortico-ponto-cerebellar circuitry, *Hox* genes

## Abstract

The pontine nuclei (PN) are the largest of the precerebellar nuclei, neuronal assemblies in the hindbrain providing principal input to the cerebellum. The PN are predominantly innervated by the cerebral cortex and project as mossy fibers to the cerebellar hemispheres. Here, we comprehensively review the development of the PN from specification to migration, nucleogenesis and circuit formation. PN neurons originate at the posterior rhombic lip and migrate tangentially crossing several rhombomere derived territories to reach their final position in ventral part of the pons. The developing PN provide a classical example of tangential neuronal migration and a study system for understanding its molecular underpinnings. We anticipate that understanding the mechanisms of PN migration and assembly will also permit a deeper understanding of the molecular and cellular basis of cortico-cerebellar circuit formation and function.

## Introduction

The basal pontine nuclei (BPN) (also known as basilar pons, pontine gray nuclei or pontine nuclei (PN)) and the reticulotegmental nuclei (RTN) (also known as nucleus reticularis tegmenti pontis) are located within the ventral portion of the pons. Both nuclei (together referred to as PN) cannot be distinguished molecularly during development. The PN constitute the main mossy fiber input to the cerebellum carrying information from the cerebral cortex. The development of the PN has been intensively studied. Considerable progress has been made in understanding how the stereotypic tangential neuronal migration and positioning of the PN next to the ventral midline of the rhombomere (r) 3- and 4-derived territory are orchestrated. Also, several studies addressed how the initial steps of axon guidance to the cerebellum and innervation from the cortex are organized. Yet, our understanding of the mechanisms that pattern the complex input-output circuitry of the PN is limited. Recent studies have shown that the PN are composed of a heterogeneous population of projection neurons and that this diversity might in turn contribute to the complex connectivity between neocortex, PN and cerebellum.

The aim of this review article is to provide an overview of our current understanding of the development of the PN and their circuitry. Moreover, we propose that developmental programs and protomaps established at the pre-migratory stage contribute in shaping the cortico-ponto-cerebellar circuitry. This is further influenced by environmental factors during migration and nucleogenesis. By summarizing the main literature that attempts to describe the complex input-output connectivity of the PN and their partially topographic organization, we describe emerging concepts on the logic behind the cortico-ponto-cerebellar connectivity. We also speculate about the evolution of PN and the cortico-ponto-cerebellar pathway. Lastly, we discuss outstanding questions and how they can be approached.

## Pontine Nuclei as Part of The Precerebellar System: Specification at The Rhombic Lip

**Figure 1:**
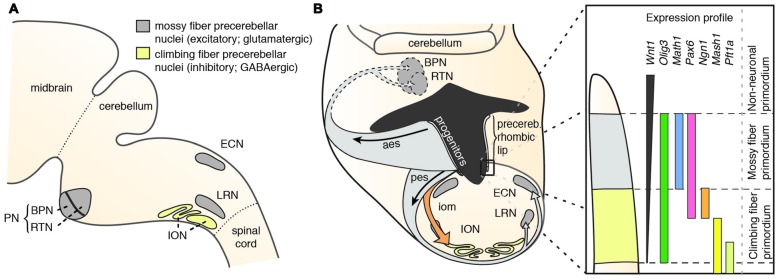
**The precerebellar nuclei and their specification. (A)** The PN comprise the BPN and RTN. They are part of the mossy fiber precerebellar nuclei that also include ECN and LRN. The precerebellar ION is the source of the climbing fibers. **(B)** The PN are derived from progenitors at the rhombic lip. The rhombic lip is dorsoventrally patterned. The PN are derived from a neuroepithelial domain that expresses *Wnt1*, *Olig3*, *Pax6* and *Atoh1*. Specified PN neurons migrate in the aes to their final position in the ventral pons. aes: anterior extramural stream, BPN: Basal Pontine Nuclei, ECN: External Cuneate Nucleus, iom: inferior olivary migratory stream, ION: Inferior Olivary Nucleus, LRN: Lateral Reticular Nucleus, pes: posterior extramural stream, PN: Pontine Nuclei (**B** is adapted with permission from Altman and Bayer, 1987d, Wiley).

Precerebellar nuclei, including the inferior olivary nucleus (ION), external cuneate nucleus (ECN), lateral reticular nucleus (LRN), RTN and BPN (Figure 1A) originate from the posterior (lower) rhombic lip, an embryonic proliferative neuroepithelium that lies in the dorsal rhombencephalon and surrounds the alar recess of the fourth ventricle (Altman and Bayer, 1987a,b,c,d). Development of rhombic lip derivatives has been intensively studied (reviewed in Di Meglio and Rijli, 2013; Sotelo and Chedotal, 2013; Hatanaka et al., 2016). A hallmark of the rhombic lip is its dorsoventrally graded expression of *Wnt1* (Rodriguez and Dymecki, 2000; Figure 1B). Precerebellar neuron progenitor pools within the *Wnt1* rhombic lip domain are molecularly and spatially defined, giving rise to distinct neuronal populations. All mossy fiber precerebellar neurons, i.e., those contributing to ECN, LRN, RTN and BPN, are derived from a defined dorsal domain of the rhombic lip specified by high *Wnt1* expression levels and the expression of the basic helix-loop-helix (bHLH) transcription factor *Atoh1* (*Math1*; Rodriguez and Dymecki, 2000; Machold and Fishell, 2005; Wang et al., 2005). Climbing fiber inferior olive neurons are instead derived from progenitors with low *Wnt1* levels that express *Ngn1* and *Pft1a* and are located ventral to the *Atoh1*-domain (Figure 1B). Consequently, *Atoh1* knockout mice lack all precerebellar nuclei except the ION (Wang et al., 2005), whereas *Pft1a* null mutants lack the ION, but not the other precerebellar nuclei (Yamada et al., 2007, 2014).

**Figure 2:**
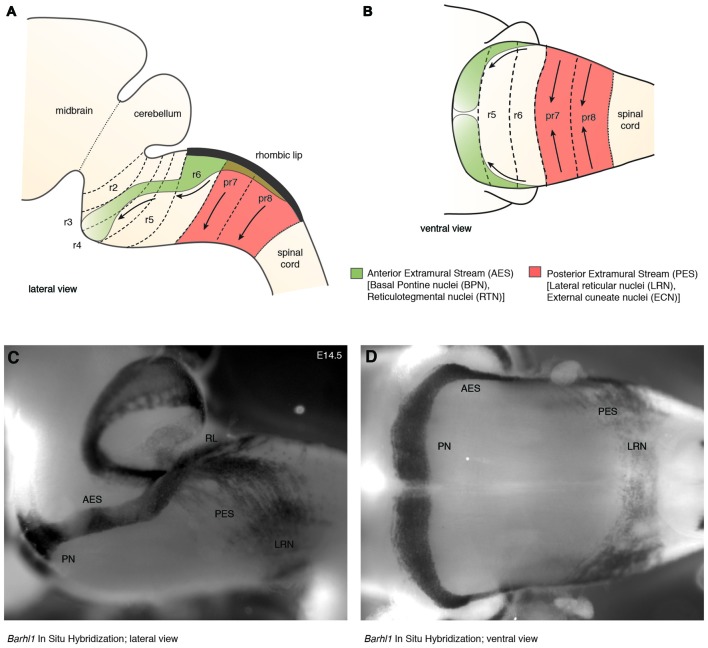
**The migratory streams of precerebellar neurons. (A–D)** The PN derive from rhombomere (r)6–pr8 precerebellar rhombic lip and take a rostroventral path in the AES to finally settle ventrally in r3 partially and in r4 derived territories. Other precerebellar nuclei such as the LRN and ECN derive from pr7–pr8, migrate ventrally in the PES, cross the midline, and settle contralaterally at more dorsal positions. Migratory streams are shown from lateral **(A,C)** and ventral **(B,D)** views as schematic drawings **(A,B)** and as whole-mount *in situ* hybridization using the precerebellar neuron marker *Barhl1*, as a probe **(C,D)**. AES: anterior extramural stream, ECN: external cuneate nucleus, LRN: Lateral Reticular Nucleus, PES: posterior extramural stream, PN: Pontine Nuclei, pr: pseudo-rhombomere, RL: Rhombic lip (**C,D** from Kratochwil, 2013).

In mouse, the posterior lower rhombic lip (precerebellar lip) spans rhombomere (r)6 to pseudo-rhombomere (pr)8 (Figures 2A,B). These rostrocaudal progenitor domains are molecularly defined by the partially overlapping expression of *Hox* genes of the paralog group 2–5 (*Hox2–5*) whereas they lack *Hox6–11* expression (Di Meglio et al., 2013; Tomás-Roca et al., 2016). More anterior *Atoh1*-positive rhombic lip progenitors generate the granule neurons of the cerebellum (r1-derived) and the neurons of the brainstem cochlear complex (r2-r5-derived), respectively (Rodriguez and Dymecki, 2000; Machold and Fishell, 2005; Wang et al., 2005; Wingate, 2005; Farago et al., 2006; Ray and Dymecki, 2009). Hence, precerebellar nuclei neurons are generated from distinct intersections of dorso-ventral (DV; *Atoh1*^+^/*Wnt1*^+^) and anterior-posterior (AP; *Hox2–5*^+^) progenitor pools, as assessed in several fate mapping studies using rhombomere- and DV progenitor-specific *Cre* and *FLP recombinase* expressing mouse lines, respectively (Rodriguez and Dymecki, 2000; Wingate, 2005; Fu et al., 2011; Di Meglio et al., 2013).

Several other transcription factors have been shown to affect the development of the PN and other precerebellar nuclei. The bHLH transcription factor *Olig3* is expressed in the rhombic lip and encompasses the *Atoh1*^+^ domain. *Olig3*^−/−^ mutants have significantly reduced levels of *Atoh1* expression and reduced PN size (Liu et al., 2008). Also, PAX6 has been shown to influence the specification of dorso-ventral domains in the rhombic lip by maintaining normal levels of BMP signaling. *Pax6* null mutants have a reduced *Atoh1* domain, but an increased *Ngn1* domain. Hence, *Pax6* mutants have strongly reduced PN, but larger ION (Engelkamp et al., 1999; Landsberg et al., 2005).

Finally, distinct precerebellar nuclei are generated during different ontogenetic periods, as shown initially by tritiated thymidine radiographic studies in the rat (Altman and Bayer, 1987d). This was confirmed by fate mapping with temporally inducible tamoxifen-dependent *Atoh1::CreER^T2^* lines (Machold and Fishell, 2005; Wang et al., 2005) and *in utero* electroporation in mice (Okada et al., 2007). The *Ptf1a*^+^ ION neurons are the first ones to be generated and to migrate (E10.5–E11.5), followed by the *Atoh1*^+^ LRN (E11.5–12.5), ECN (E11.5–12.5), RTN (E12.5–E13.5) and lastly the BPN (E13.5–E16.5) (Pierce, 1966; Altman and Bayer, 1987d; Machold and Fishell, 2005; Wang et al., 2005; Okada et al., 2007).

## A Long Way to Go: The Tangential Migration of Pontine Nuclei Neurons

Rhombic lip derivatives including the PN are amongst the best studied examples of tangential migration (Hatten, 1999; Nóbrega-Pereira and Marín, 2009; Di Meglio and Rijli, 2013; Sotelo and Chedotal, 2013; Hatanaka et al., 2016). From the rhombic lip, PN neurons undertake a long-distance tangential migration via the anterior extramural stream (AES). In contrast to radial migration, where neurons use radial glia as a scaffold, AES tangentially migrating neurons move orthogonally to the orientation of radial glia, just beneath the meninges.

**Figure 3:**
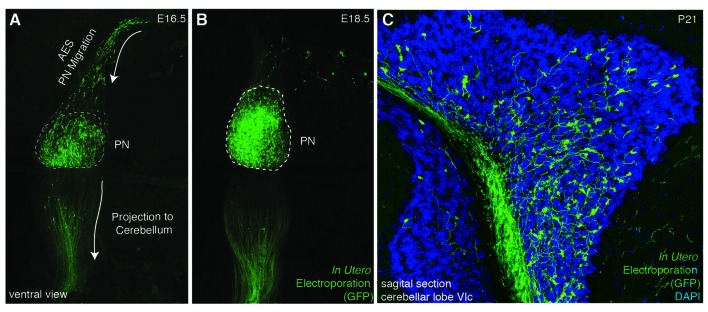
***In utero* electroporation as a tool for analyzing PN development**. **(A–C)** *In utero* electroporation of E14.5 lower rhombic lip with *pCAG-eGFP* allows the visualization of the AES **(A)**, PN assembly **(A,B)**, and their axon development and projection to the cerebellum **(B,C)**. AES: anterior extramural stream, PN: pontine nuclei. (**A–C** from Kratochwil, 2013).

**Figure 4:**
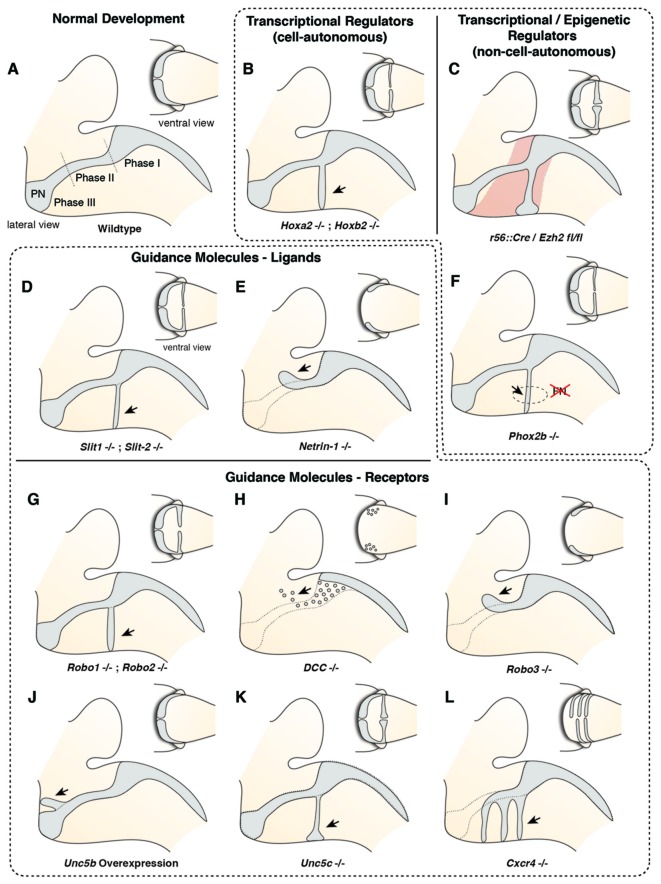
**Molecular mechanisms controlling migration of pontine neurons (I)**. **(A)** The migration of PN neurons can be divided into three phases (Phase I–III). In the first phase (phase I) PN neurons migrate ventrally, switch then to a rostral direction (phase II), and finally migrate to the ventral midline (phase III). **(B–L)** Several cell-autonomous **(B,G–L)** and non-cell autonomous **(C–F)** factors including guidance molecules **(D,E,G–L)** and upstream epigenetic **(C)** and transcriptional regulators **(B,F)** have been shown to influence all or distinct phases of pontine neuron migration. PN: pontine nuclei.

PN neurons migrate rostroventrally (Figures 1–4), unlike other precerebellar neurons that directly take a ventral route from their dorsal progenitor zone (Figures 1, 2). Migration of PN neurons can be subdivided into three phases (Figure 4A; Geisen et al., 2008). After leaving r6–pr8, PN neurons migrate ventrally (phase I). Next, they turn rostrally and migrate through r5 and r4, passing the vestibulocochlear and facial nerve roots (phase 2). The migratory stream then enters r3 and reaches the trigeminal nerve root in the caudal aspect of r2 where it turns again ventrally (phase 3) and finally settle on both sides of the floor plate in-between rostral r3 and rostral r5 derived territories (Farago et al., 2006; Geisen et al., 2008). Some PN neurons cross the midline and contribute to the contralateral PN. In mice, the generation, migration, and settlement at final destination of PN neurons takes place between E13.5 and E18.5 (Okada et al., 2007; Hatanaka et al., 2016). The migration time of a single neuron from leaving the rhombic lip to reaching its final destination is approximately two days with the last neurons arriving at E18 (Okada et al., 2007; Hatanaka et al., 2016).

Several reasons make the precerebellar system a suitable system for studying tangential migration. First, the subpial migratory streams can be easily visualized. Cells migrate directly underneath the meninges. Several markers allow to specifically distinguish migrating precerebellar cells from the surrounding tissue, including *Barhl1* (Figures 2C,D) (*Mbh2*) (Li et al., 2004), *Pax6* (Engelkamp et al., 1999), and *Tag1* (Backer et al., 2002) permitting the visualization of the complete migratory pathway on whole-mount preparations. Using this approach, it is also possible to efficiently screen for migratory defects in knockout mutant mice. Also, several *Gfp* and *Cre* or *Flp* recombinase transgenic and knock-in mouse lines are available that target precerebellar neurons facilitating not only the analysis of the migration phenotypes but also allowing conditional knockout targeting of precerebellar neurons (Danielian et al., 1998; Dymecki and Tomasiewicz, 1998; Rodriguez and Dymecki, 2000; Machold and Fishell, 2005; Wang et al., 2010; Di Meglio et al., 2013; Lewis et al., 2013; Kratochwil and Rijli, 2014). More recently, *in utero* electroporation of precerebellar progenitors has become a powerful tool for analyzing the molecular mechanisms of tangential migration (Okada et al., 2007; Watanabe and Murakami, 2009; Di Meglio et al., 2013; Kratochwil, 2013; Zelina et al., 2014). Using this approach, not only can migrating neurons be effectively visualized at a single cell resolution by reporter gene expression, but they can also be co-electroporated with genes of interest or RNA interfering constructs for gain-of-function or knockdown experiments, respectively. This approach has the advantage that the behavior of the electroporated cells can be analyzed in a wild type environment (Figure 3).

## Molecular Mechanisms Controlling The Migration of Pontine Nuclei Neurons

**Figure 5:**
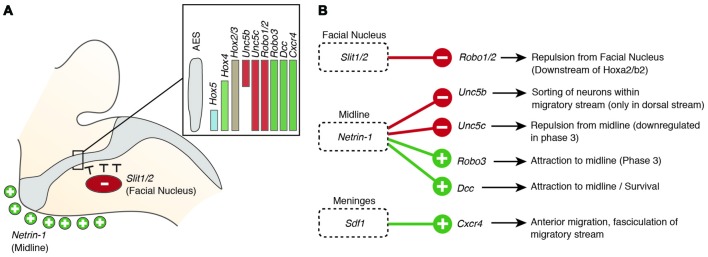
**Molecular mechanisms regulating the migration of pontine neurons (II)**. **(A,B)** Several genes including ligand guidance molecules and their receptors regulate the migration of PN neurons including *Slit1/2*, *Robo1/2*, *Rig1/Robo3*, *Netrin-1*, *Dcc*, *Sdf1* and *Cxcr4*. **(A)** Slits, released from the facial nucleus, repel rostrally migrating PN neurons, which express the receptors *Robo1* and *Robo2*. *Robo2* expression is regulated by the *Hox*2 genes, *Hoxa2* and *Hoxb2*. Ventral migration of PN neurons is guided by the ligand Netrin-1 that is released from the midline. **(B)** A balanced expression of *Dcc* and *Rig1/Robo3*, mediating attraction and *Unc5b/c*, mediating repulsion, controls the targeting to the ventral midline. The *Unc5b* receptor is expressed in the dorsal part of the migratory stream **(A)**, contributing to maintain the position of r6 derived PN neurons. *Hox* genes display dorsoventrally nested expression within the migratory stream **(A)** *Hox5* genes downregulate *Unc5b* expression in the ventral part of the migratory stream. AES: anterior extramural stream, PN: pontine nuclei.

Several guidance factors and transcription factors have been shown to regulate the complex migratory pathway of PN neurons (Figures 4, 5). PN neurons, as other migrating precerebellar nuclei neurons, are attracted by the floorplate. This attraction is mediated by NTN1/DCC signaling, with *Ntn1* being expressed in the floorplate and the *Dcc* receptor in migrating precerebellar neurons (Fazeli et al., 1997; Yee et al., 1999; Alcántara et al., 2000; Zelina et al., 2014). In *Dcc* and *Ntn1* null mutants, PN neurons do not reach the midline and are stalled in a medio-lateral position (Figures 4E,H; Yee et al., 1999; Zelina et al., 2014). Furthermore, cell number is decreased in both mutants, in accordance with the known role of NTN1/DCC as survival factors (Llambi et al., 2001).

But what makes PN neurons migrating rostrally? By the time the PN neurons migrate, most other precerebellar nuclei have already reached their final destination or are in the last phases of migration. Also, other hindbrain nuclei including the facial motor nucleus (FN) have already formed. The FN is a source of the repulsive diffusive guidance molecules SLIT2 and SLIT3 (Geisen et al., 2008). The FN neurons are generated in r4 and migrate tangentially to ventral r6 (Garel et al., 2000). Once the FN settles in the ventral r6 derived territory it repels migrating PN neurons that express the SLIT receptors ROBO1 and 2 and switch from phase 1 (ventral migration) to phase 2 (rostral migration; see above) (Di Meglio et al., 2008; Geisen et al., 2008). The expression of *Robo2* is regulated by the *Hox2* paralogs, *Hoxa2* and *Hoxb2* (Geisen et al., 2008). Moreover, in *Hoxa2;Hoxb2* knockout mice (Figure 4B) PN neurons migrate prematurely to the ventral midline, similar to *Robo1;Robo2*, *Slit1;Slit2* and *Robo2;Slit2* mutant mice (Figures 4D,G; Di Meglio et al., 2008; Geisen et al., 2008). Ectopic PN neuron migration could also be observed in *Phox2b* mutant mice that do not develop the Slit1/2 expressing facial nucleus (Figure 4F; Geisen et al., 2008). Once beyond the influence of the FN, PN neurons are guided by Ntn1/Dcc signaling to their final destination lateral to the floorplate.

In addition to NTN/DCC and ROBO/SLIT signaling, several factors are involved in PN neuron migration. In mutants for *Unc5c*, a repulsive receptor of NTN1, PN neurons migrate ectopically (Figure 4K). While their ventral positioning is unaffected, some neurons migrate prematurely towards the midline at ectopic posterior positions. These defects can be rescued by overexpression of *Unc5c* in PN neurons suggesting a cell-autonomous role (Kim and Ackerman, 2011). The paralog *Unc5b* is also expressed in migrating PN neurons, however only in a subset. Overexpression of *Unc5b* results in anterior ectopic migration as well as lateral migratory arrest (Figure 4J; Di Meglio et al., 2013). A second repulsive guidance protein, DRAXIN, which interacts with DCC (Islam et al., 2009), was also suggested to play a role in PN neuron migration. Although DRAXIN inhibits precerebellar neuron migration *in vitro*, no significant differences in PN neuron migration could be shown in *Draxin* knockout animals (Riyadh et al., 2014). DCC also cooperates with RIG1/ROBO3 to mediate NTN1 attraction (Zelina et al., 2014). In *Robo3* deficient mice, PN neurons fail to arrive at the midline and are instead arrested at a lateral position after phase 2 of migration, forming a disorganized ectopic cluster of cells (Figure 4I; Marillat et al., 2004; Zelina et al., 2014). The phenotype is reminiscent of the *Dcc* and *Ntn1* knockouts, however without a clear decrease in cell number. In summary, these results suggest that NTN1 and ROBO signaling are integrated in a complex manner throughout the migration of PN neurons. The ratio of repulsion vs. attraction is tightly regulated and balanced, resulting in the stereotypic migration along the AES.

Several other factors have been shown to influence PN neuron migration. In mouse mutants for the glycosyltransferase *LARGE*, the migration is stalled after phase 2 resembling the phenotype in *Robo3* mutants (Qu et al., 2006). Similarly, knockdown of *Calmodulin (Calm1)* resulted in aberrantly positioned PN neurons, and additionally nucleogenesis was affected (Kobayashi et al., 2015). Cadherins, a group of adhesion molecules involved in collective cell migration (Theveneau and Mayor, 2012), have been shown to regulate the tangential migration of precerebellar neurons (Taniguchi et al., 2006). Lastly, the meninges are also involved in guiding migrating PN neurons (Zhu et al., 2009). The chemokine SDF1, ligand of the CXCR4 receptor is released from the meninges and is required for the marginal migration of the PN neurons directly beneath the meninges. Removal of the meninges or *Cxcr4* knockout induced submarginal migration and a far less confined migratory stream (Figure 4L; Zhu et al., 2009). Additionally, SDF1/CXCR4 signaling might contribute to the anterior migration of pontine neurons. *Cxcr4* null mutants show multiple ectopic posterior pontine clusters (Zhu et al., 2009). Therefore, the SDF1/CXCR4 signaling pathway might either have an instructive role (*Sdf1* has higher expression levels anteriorly) and/or modulate the responsiveness of pontine neurons to other anterior guidance cues such as Netrins and Slits. Similarly, retinoic acid (RA) is also released from the meninges and increased RA levels have been suggested to induce defasciculation of the migratory stream and posterior ectopic migration (Yamamoto et al., 2003, 2005). However, due to the extensive effects of varying RA levels on several developmental processes, direct roles in PN guidance are difficult to pinpoint. Hence, SDF1 might not be the only non-cell-autonomous instructive signal from the meninges contributing to the migration of pontine neurons.

But how is the expression of these guidance factors controlled? Besides the aforementioned transcription factors such as ATOH1 and PAX6, several other transcription factors have been shown to be not only essential for proper migration but also for PN neuron specification. HOXA2 and HOXB2 act upstream of *Robo2* in migrating PN neurons regulating their response to repulsion from the FN (Geisen et al., 2008), resulting in the rostroventral migratory route. The helix-loop-helix transcription factors NSCL-1 and NSCL-2 are strongly expressed in the AES (Schmid et al., 2007). Single null mutants have no PN neuron migration defects, whereas in *Nscl-1/Nscl-2* compound mutants the basal PN are absent and the RTN are strongly reduced (Schmid et al., 2007). *Dcc* was downregulated in *Nscl-1/Nscl-2* double mutants, suggesting that NSCL-1/NSCL-2 act upstream of *Dcc*, possibly explaining the increased apoptosis in double mutants. In mutants for the Nuclear Factor Ib (*Nfib*), the PN are greatly reduced and the migration is delayed, suggesting that early migrating PN neurons are more vulnerable to the knockout of *Nfib*. However, their targeting to the ventral pons is not disturbed (Kumbasar et al., 2009). Additionally, genes that are involved in post-transcriptional regulation have been proposed to control migration of precerebellar neurons including the RNA-binding protein *Csde1* for the PN (Kobayashi et al., 2013) and *Musashi1* (*Msi1*) for LRN and ECN (Kuwako et al., 2010).

## Nucleogenesis and Patterning of The Pontine Nuclei

The migration of PN neurons from lower rhombic lip to their target region in the ventral pons is followed by nucleogenesis (Altman and Bayer, 1987d; Kawauchi et al., 2006). During this process PN neurons form a 3-dimensional aggregate bulging out of the ventral pons. In mice, PN nucleogenesis occurs between E14.5, when the first neurons arrive at their final destination, and E18.5, when nucleogenesis is close to completion (Shinohara et al., 2013). Various studies done in rodents provide a detailed analysis of the movement of neurons during nucleogenesis; however, the molecular mechanisms involved in the regulation of this process are poorly understood.

**Figure 6:**
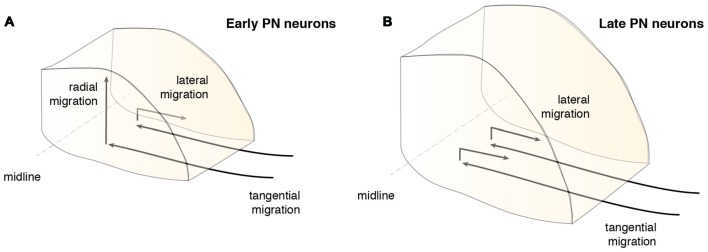
**Migration modes during PN assembly**. After the tangentially migrating PN neurons arrive at the ventral midline, they switch their migratory mode and/or direction. **(A)** Early arrived PN neurons partially migrate radially contributing to the core of the PN, including the neurons of the RTN, or switch their migration laterally below the surface (Shinohara et al., 2013). **(B)** Late PN neurons rarely migrate radially, but mostly migrate laterally and stack onto progressively forming layers of PN neurons and therefore mainly contribute to the BPN (Shinohara et al., 2013). BPN: basal pontine nucleus, PN: pontine nuclei, RTN: reticulotegmental nucleus.

**Figure 7:**
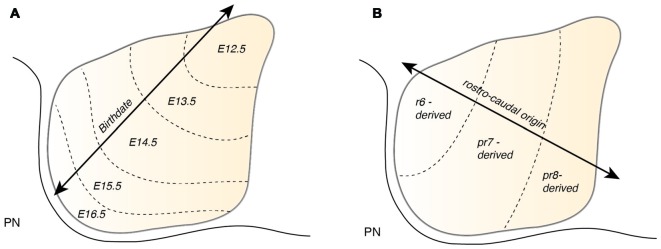
**Patterning of the PN during nucleogenesis**. **(A)** During nucleogenesis, PN neurons populate the target region in an inside-out (dorsoventral) fashion with early born neurons building the inner core of the PN and late born neurons contributing to the outer shell of the PN. **(B)** The rostrocaudal order of PN neuron progenitors at the lower rhombic lip is maintained by postmitotic PN neurons during tangential migration and nucleogenesis. r6 derived neurons position themselves in the most rostral part of the PN whereas pr8 derived neurons settle in the most caudal part of the PN. PN: pontine nuclei, r: rhombomere, pr: pseudo-rhombomere.

Timing plays an important role during PN nucleogenesis. Early born-early arriving neurons switch their migration mode from tangential to radial near the ventral midline (Watanabe and Murakami, 2009; Shinohara et al., 2013). Depending on the cellular behavior of neurons, this switch can be categorized into two types (Figure 6). The first category comprises neurons that pause their migration at the ventral midline and switch to radial migration (Figure 6A). Hereby, the soma of these neurons migrates orthogonal to the surface, leaving the leading process behind. The second category is composed of neurons that change their migratory direction without ceasing migration (Watanabe and Murakami, 2009). In both categories, migrating neurons grow a new short process or a bifurcation of the leading process, enabling the neuron to take up a new migratory route. This switch between tangential to radial migration is most apparent at E15.5 (Kawauchi et al., 2006). Interestingly, the ability to switch between tangential to radial migration was only seen in early born-early arriving PN neurons (Watanabe and Murakami, 2009; Shinohara et al., 2013). Late born PN neurons instead stack ventrally to early arriving neurons (Altman and Bayer, 1987d; Shinohara et al., 2013) resulting in an inside-out lamellae-like structure of the PN (Figures 6B, 7A). Because early born PN neurons are able to migrate dorsally and late born PN neurons are mostly located more ventrally, it was hypothesized that the RTN is populated mostly by early born neurons, whereas BPN is formed of both early and late born PN neurons (Altman and Bayer, 1987d; Shinohara et al., 2013).

In addition to the migration along the dorsoventral axis, migrations along the mediolateral and rostrocaudal axes can also be observed during PN nucleogenesis (Shinohara et al., 2013; Figure 6). Neurons enter the forming PN and migrate medially. However, some neurons migrate laterally (lateral migration), settling at a more distal position, eventually resulting in an expansion of the PN along the mediolateral axis. Both early born and late born PN neuron subsets can switch from medial to lateral migration. Migration along the rostrocaudal axis of the forming PN occurs rarely and mostly involves early born neurons. In summary, distinct neuron migratory patterns contribute to PN nucleogenesis, expansion and organization. The switch from tangential to radial migration contributes to nucleogenesis along the dorsoventral axis, whereas a switch from medial to lateral migration contributes to mediolateral expansion. Lastly, widening of the tangential migratory stream has a direct bearing on PN rostrocaudal expansion (Shinohara et al., 2013).

A few studies have investigated the molecular regulation of PN nucleogenesis and the maintenance of PN integrity. The transmembrane immunoglobulin superfamily molecule NEPH2 regulates the movement of neurons within the PN (Nishida et al., 2011). *Neph2* null mutants show disrupted intranuclear migration of PN neurons along the mediolateral axis. Most neurons in *Neph2* mutant are found to be stuck along the ventral midline. In mice lacking the homeobox transcription factor BARHL1/MBH2, PN are only a third of their normal size. *Barhl1* is expressed in all rhombic lip derivatives except inferior olivary nuclei throughout migration and nucleogenesis. The phenotype of *Barhl1* null mutants appears to be mainly due to a postnatal increase in apoptosis (Li et al., 2004). However, the downstream mechanisms are still unclear.

The process of PN nucleogenesis occurs primarily prenatally; yet, there is a dramatic increase in the PN size postnatally. The peak of postnatal growth of PN is between postnatal day 0 (P0) and P4. Interestingly, this growth is mainly a consequence of a large production of oligodendrocytes from the *Sox2^+^/Olig2^+^* expressing progenitors present in the ventricular zone along the fourth ventricle, the midline domain and in the parenchyma (Lindquist et al., 2016).

## One Stream to Bring Them All—The Cryptic Heterogeneity of The Anterior Extramural Stream

*Atoh1*-derived PN neurons migrate along the AES as a large, seemingly homogeneous, stream of cells. However, the AES may be composed of subsets of neurons bearing heterogenous positional cell identities. Several factors may generate distinct subsets of PN neurons. One important factor of heterogeneity is their birthdate (Altman and Bayer, 1987d). As noted in the previous section, PN neurons are generated between E12.5 and E16.5 and nucleogenesis occurs until E18.5 (Figure 7A). The timing of birth of a PN neuron defines its position along the dorsoventral axis (Altman and Bayer, 1987d), hence providing a source of distinct PN neuron subpopulation positional identity.

**Figure 8:**
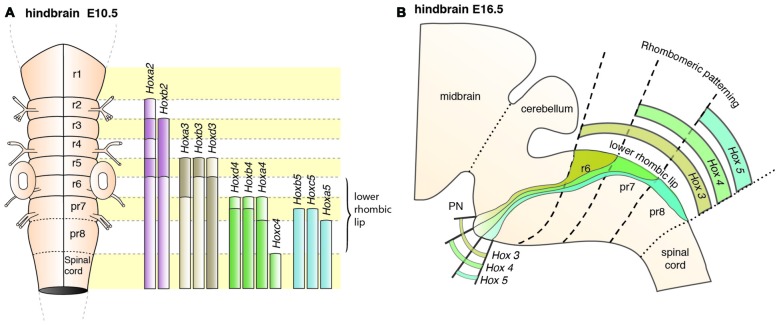
**Patterning of PN progenitors along the rostrocaudal axis. (A)** The progenitors of the PN in the lower (caudal) rhombic lip are generated in r6, pr7 and pr8 and are molecularly defined by the expression of the *Hox* genes. r6 derived PN neurons express members of the *Hox2* and *Hox3* paralog group, pr7 derived neurons express *Hox2–4* genes and pr8 derived neurons *Hox2–5* genes. **(B)** *Hox* expression is maintained throughout migration and PN assembly. Moreover, PN neuron subsets originated from r6 or pr7–8 remain segregated and keep their relative position throughout tangential migration and settling in the forming PN, with r6-derived neurons being positioned dorsally in the stream and rostrally in the mature PN. PN: pontine nuclei, r: rhombomere, pr: pseudo-rhombomere.

PN neurons are also segregated into distinct subpopulations settling at different positions along the rostrocaudal axis of the forming nuclei (Di Meglio et al., 2013). PN neurons originate from r6–pr8 (Figure 8). Subsets of pontine neurons derived from different anteroposterior (AP) progenitor domains do not move freely in the migratory stream nor in the forming PN, but tend to remain dorsoventrally segregated during AES migration and settle in distinct anteroposterior domains in the PN (Figures 7B, 8B). This was shown by fate mapping approaches using rhombomere specific Cre-expressing mouse drivers crossed with Cre-dependent floxed reporter lines (Di Meglio et al., 2013). Pontine neuron subsets derived from different AP rhombic lip progenitor domains also exhibit distinct expression profiles of HOX2–5 transcription factors. This molecular information is maintained throughout their tangential migration and nucleogenesis (Figures 7B, 8). Pr8 derived neurons express *Hox5* genes, namely *Hoxa5*, *Hoxb5* and *Hoxc5*. *Hox5* expressing PN cells leave the rhombic lip at posterior positions, migrate in the most ventral part of the AES, and finally settle in the most posterior part of the nuclei (Di Meglio et al., 2013). But does the differential expression of *Hox* genes influence pontine neuron migratory behavior and/or PN circuit formation?

In compound *Hoxa5;Hoxb5;Hoxc5* null mutants, high levels of *Unc5b* are expressed throughout the AES (Di Meglio et al., 2013). *Unc5b* is highly expressed in r6 derived PN neurons, but has low expression in pr8 derived PN neurons (Figure 5A). The lack of this expression gradient in compound *Hox5* knockout mice thus suggests that *Hox5* genes normally inhibit *Unc5b* expression in the ventral AES (Di Meglio et al., 2013). Moreover, *Unc5b* overexpression results in anterior ectopic migration and stalled migration lateral to the normal nucleus (Di Meglio et al., 2013). Additionally, overexpression of *Netrin* *1* (*Ntn1*) in the r5 and r6 derived migratory environment of the AES leads to premature migration of *Unc5b* negative cells to the midline generating ectopic PN (Di Meglio et al., 2013). Differential *Hox* expression in the AES may therefore provide PN subsets with distinct UNC5B-dependent responses to environmental guidance cues (NTN1) and thus contribute to maintaining neuronal position during migration and nucleogenesis (Figure 4). Interestingly, *Ntn1* expression is epigenetically controlled (Di Meglio et al., 2013). Transcriptional silencing mediated by the polycomb protein EZH2 maintains the ventral restriction of *Ntn1* expression at the midline. Loss of *Ezh2* results in spatial expansion of the *Ntn1* expression domain and ectopic migration of *Unc5b* negative cells (Figure 4C, Di Meglio et al., 2013).

In summary, migrating PN neuron subsets are regulated through a tightly balanced mix of repulsive and attractive cues. Anterior, r6 derived, PN neurons migrating in the dorsal part of the AES lack *Hox4* and *Hox5* expression and display high expression levels of *Unc5b*, thus resulting in stronger repulsion from the midline than *Hox4*^+^;*Hox5*^+^;*Unc5b*^low^ expressing neurons in more ventral aspects of the AES (Di Meglio et al., 2013). If such a tightly balanced signaling system is challenged through genetic manipulations, three distinct phenotypes can be observed in which PN neurons are derailed from their stereotypic migratory pathways. Neurons can either: (a) prematurely migrate ectopically and take a more posterior pathway; or, (b) be stalled generating an ectopic cluster lateral to their normal position; or, (c) migrate ectopically towards a more anterior position. It is predicted that for (a) mostly pr8 derived *Hox5* expressing, ventrally migrating neurons are affected, while for (b) and (c) mostly dorsally migrating, *Hox5* negative neurons are affected (Di Meglio et al., 2013). Also, reduction of PN size observed in several mutant lines might in fact be due to selective loss of rostrocaudal (or early/late born) PN neuron subsets.

## Molecular Determinants of Pontine Nuclei Connectivity

At the same time when PN neurons reach their final position, axonal branches project towards the cerebellum. Mossy fiber afferents from the PN will eventually synapse with the granule cells. Several cell- and non-cell-autonomous molecular factors have been identified in rodents to regulate PN neuron axon growth, target selection and synapse formation.

Most of the mossy fibers emerging from BPN neurons target the contralateral cerebellum. The position of the neurons within the BPN is thought to be a determinant of the laterality of axonal projections (Cicirata et al., 2005). *ZIC1*, a zinc finger transcription factor expressed in mossy fiber BPN neurons was identified to regulate BPN neuron position as well as axon laterality (DiPietrantonio and Dymecki, 2009). In *Zic1* mutants, a higher number of mossy fiber afferents innervated the ipsilateral cerebellum, thus suggesting a role of *ZIC1* in self autonomously regulating the laterality of BPN mossy fibers (DiPietrantonio and Dymecki, 2009). At high expression levels, *ZIC1* may act to suppress activity of RIG1/ROBO3, an axon guidance molecule regulating laterality of axonal projections (Renier et al., 2010) in addition to regulating neuronal migration (see above). Moreover, several axon guidance molecules are implicated in the pathfinding of axons to the cerebellum. The previously discussed UNC5C receptor controls not only migration but also axon guidance of PN fibers to the cerebellum. PN projections of *Unc5c* mutants turn caudally or rostrally instead of directly projecting laterally towards the cerebellum (Kim and Ackerman, 2011). Additionally, the axon guidance molecule, semaphorin 3A (SEMA3A) and its receptor component neuropilin-1 *(NPN-1)* are potentially involved in selective targeting of the BPN neurons in the cerebellum (Solowska et al., 2002). BPN neurons express *Npn-1*, while *Sema3A* is expressed in the cerebellum. However, the expression of both *Npn-1* and *Sema3A* varies across the BPN and in different cerebellar regions or lobules, respectively. Due to varying expression of *Npn-1* along the rostrocaudal axis of the BPN, a graded repulsive responsiveness to SEMA3A was identified in the BPN, with higher levels of responsiveness to SEMA3A rostrally as compared to caudally. Thus, high Npn-1 expressing BPN mossy fibers may selectively avoid high Sema3A expressing cerebellar lobules resulting in topographic connectivity of BPN axons. Upstream of this graded expression could be the *Hox* genes that have nested expression patterns within the PN and could have a similar role as upstream regulators in the PN as shown in other hindbrain nuclei (Gavalas et al., 1997; Oury et al., 2006; Bechara et al., 2015).

In a gene expression analysis of BPN neurons during development of the pontocerebellar mossy fibers, markers of axon elongation (for example, GAP43) were downregulated during early postnatal period and there was a simultaneous upregulation of synaptic markers (Díaz et al., 2002). The upregulation of synaptic markers is induced by interaction of mossy fibers with granule cells in the cerebellar cortex. In BPN neurons of the *weaver* mouse mutant, synaptic marker genes fail to upregulate (Díaz et al., 2002).

A key aspect of synapse formation is the regulation of axonal growth and arborization. As the axons reach their targets, the growth of axon should be regulated. In the pontocerebellar system, *Cadherin7* is expressed in both granule neurons and mossy fiber pontine neurons, regulates axonal growth when mossy fibers reach the granule layer and also initiates synapse formation (Kuwako et al., 2014). Upon downregulation of *Cadherin7*, mossy fibers fail to stop at the inner granule cell layer and instead extend up to the molecular layer where they synapse with the Purkinje cells. Neuregulins, a group of signaling proteins having known roles in development and maintenance of the nervous system have also been implicated in the maturation of pontocerebellar afferents. Neuregulins, specifically NRGbeta1, are expressed at the synapses between mossy fibers and granule cells (Ozaki et al., 2000). The expression of membrane-anchored forms at the synapse suggests a role in junction formation.

Several other factors control growth, survival and differentiation of synapses within the granule cell layers. Among the first factors shown to influence the development of pontocerebellar mossy fibers are the neurotrophic factors BDNF and NT4/5 that increase survival and collateralization of BPN projections in rodents *in vitro* (Rabacchi et al., 1999a). WNT-7a is another signaling molecule released from cerebellar granule cells that controls mossy fiber development. WNT-7a is involved in mossy fiber synapse maturation (Hall et al., 2000). WNT-7a induces mossy fiber axonal remodeling, a process characterized by shortening of axons, axonal branching and increase in growth cone size, and synapsin 1 clustering in the mossy fiber terminals. Thus WNT-7a acts as a synapse-initiating factor in the pontocerebellar mossy fiber maturation.

Purkinje cells that are positioned adjacent to the granule cell layer play an important role for the development of the pontocerebellar fibers. Interestingly, during early postnatal periods, mossy fibers transiently contact Purkinje cells. However as development proceeds, these synapses are eliminated. Several studies have identified molecular mechanisms regulating the growth of mossy fibers at the level of Purkinje cells. BMP4, a patterning molecule, is expressed in the Purkinje cells during early postnatal periods. In Purkinje cells in which BMP4 was conditionally deleted, the elimination of mossy fiber-Purkinje cell contacts were decreased by half suggesting a retrograde role of BMP4 in target specificity of the mossy fiber afferents (Kalinovsky et al., 2011). SEMA3A (also referred to as COLLAPSIN-1 or SEMAD) is another molecule expressed in cerebellar Purkinje cells. *In vitro* studies performed in chick and mouse indicated that SEMA3A, prevents mossy fiber afferents from innervating Purkinje cells by initiating collapse of growth cones (Rabacchi et al., 1999b). This effect of SEMA3A was restricted to only mossy fiber growth cones and no effect was seen on the climbing fibers (Rabacchi et al., 1999b).

## Function and Basic Connectivity of The Pontine Nuclei

The PN receive their major input from the cerebral cortex and most prominently project as mossy fibers to the granule cell layer of the cerebellum. Consequently, the PN have received most attention for their integral position in the cerebro-cerebellar communication. PN are hypothesized to serve as a first integrator of the information from cortical regions and adapt these signals for the use of the cerebellum (Schwarz and Thier, 1999). They are part of several closed loop systems, including the corticopontine–cerebellar–thalamic loop (Apps and Hawkes, 2009). Additionally, the PN not only project to the cerebellum, they also exhibit reciprocal connections with the deep cerebellar nuclei. Simplified models of PN function have suggested that PN neurons receive an “efference copy” of motor commands and process this information for the use of the cerebellum. In the cerebellum, motor plan (also referred to as “internal model”), sensory feedback, and actual performance are compared and eventual arising discrepancies are fed back to the cortex to modify further motor movements (Ito, 2008; Grimaldi and Manto, 2012). This provides a functional framework for complex motor behaviors, where sequences of precise muscle contractions have to be executed. Also it provides the structural basis of motor learning, during which cerebellar circuits are modified.

## The Topography of The Cortico-Pontine Projection: How Much of The Cortical Organization Is in The Pontine Nuclei?

**Figure 9:**
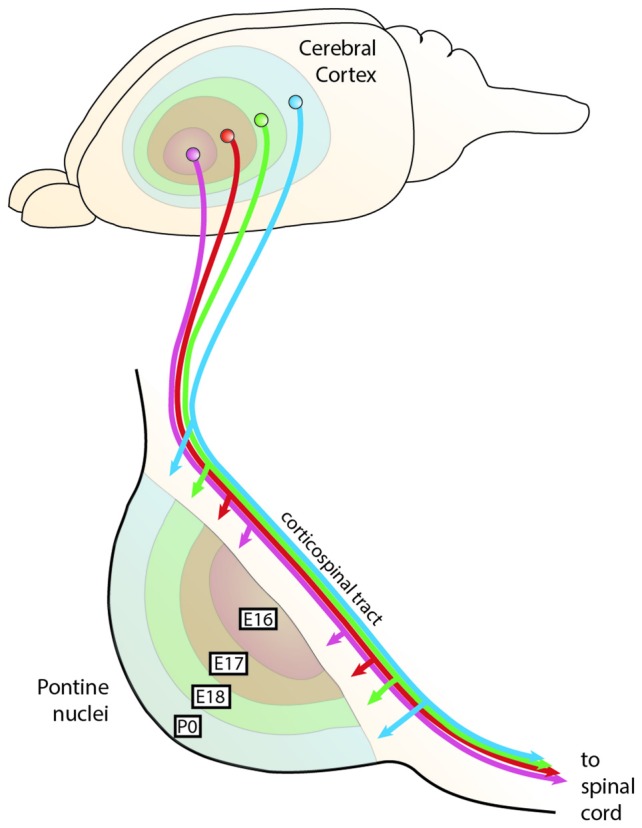
**The chrono-architectonic hypothesis of cortico-pontine circuit development**. During development, PN neurons settle in the PN in a shell-like fashion according to their birthdate. Early born neurons form the central core of the PN, later born neurons consecutively settle around the earlier born neurons forming concentric rings (Altman and Bayer, 1987d). Similarly, it has been suggested that collaterals of the corticospinal axons innervate the PN topographically at early postnatal stages in a shell-like fashion (Leergaard et al., 1995). This suggests that the birthdate of PN neurons can be linked to both nucleogenesis and spatial organization of cortical inputs (Altman and Bayer, 1997). Consequently, intrinsic differences in PN neurons born at different stages could have an instructive role for patterning the architecture of the PN as well as their innervation. PN: pontine nuclei. (Adapted with permission from Leergaard et al., 2000, Wiley).

**Figure 10:**
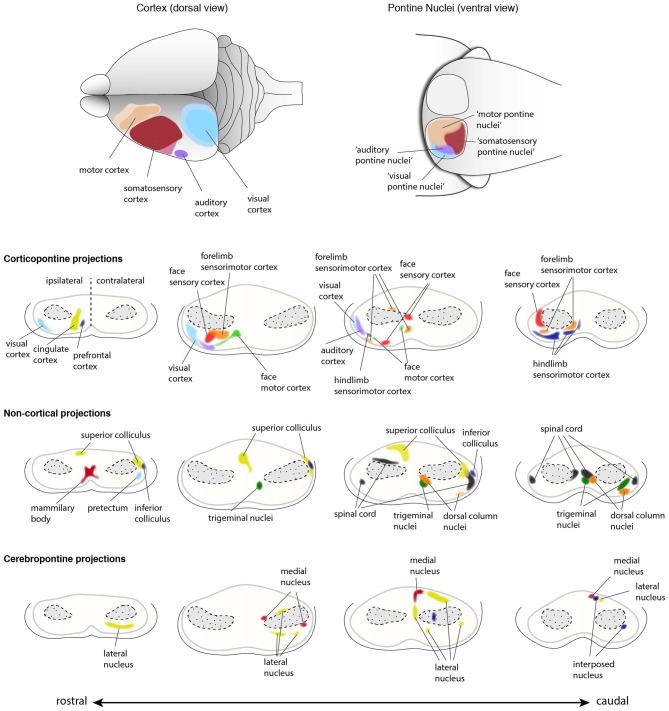
**Input innervations of the PN**. PN receive mainly projections from the cortex but also from subcortical regions. Using dye tracings, innervation patterns from various cortical and subcortical areas were identified. Cortical projections mainly innervate the ipsilateral PN, while subcortical projections innervate both ipsilateral and contralateral PN. The topography of cortical regions is roughly maintained in the PN (top panel), with auditory and visual cortex projecting to the dorso-lateral parts of the PN, motor cortex projecting to the rostral and medial areas and somatosensory cortex projecting to the caudal regions of the PN (Brodal, 1968; Leergaard et al., 1995, 2000, 2004, 2006; Leergaard, 2003; Odeh et al., 2005; Leergaard and Bjaalie, 2007). However, projections from distinct cortical areas may also partially overlap in the PN. Subcortical structures including superior and inferior colliculus, spinal cord, trigeminal nuclei and pretectum, and cerebellar nuclei also project to the PN (Kosinski et al., 1986). Projections from cortex and subcortical regions representing similar information often target the same PN region. (PN: pontine nuclei. Adapted with permission from Paxinos, 2014, Elsevier).

The majority of PN afferents arise in the cerebral cortex. Axons of cortical layer V neurons project through the cortico-fugal/cortico-spinal tract onto the PN (Figures 9, 10). In rodents, this innervation is formed shortly after birth. Two days after the corticospinal tracts bypass the developing PN, fibers or collaterals from these axonal tracts start growing into the PN forming corticopontine fibers (Leergaard et al., 1995). Several experiments suggest that the innervation is triggered by the PN neurons, since ectopically migrating neurons are innervated as well (Zhu et al., 2009; Di Meglio et al., 2013).

Both BPN and RTN receive afferents from layer V neurons of the ipsilateral cortex. However, the cortical innervation of BPN is more prominent, compared to that of RTN. Several subcortical inputs to the PN have been described. These include inputs from the spinal cord, the superior and inferior colliculus, the mammillary body, the trigeminal nuclei and the dorsal column nuclei just to name a few (summarized in Paxinos, 2014). The cortical innervation of the PN has been studied intensively. Fibers originate from sensory (somatosensory, visual, and auditory) and motor cortices with additional contributions from the caudal temporal and perirhinal cortex (Cicirata et al., 2005). The RTN predominantly receive afferents from the cingular cortex with fewer contributions from the motor and sensory cortices. Interestingly, less overlap is observed in the cortical afferents terminating in the BPN, whereas the extent of overlap in the RTN is higher, which suggests stronger convergence of information from different cortical areas in the RTN (Brodal and Brodal, 1971).

A remarkable feature of the corticopontine projections is the precise pattern of their termination in the PN. Focal tracer injections in the cerebral cortex helped in delineating the projection pattern of corticopontine projections in rodents. An at least partially topographic organization could be shown for the pathway from cerebral cortex to the pons (Brodal, 1968; Leergaard et al., 1995, 2000, 2004, 2006; Leergaard, 2003; Odeh et al., 2005; Leergaard and Bjaalie, 2007). Distinct regions in the cortex tend to project to relatively segregated regions in the PN, with only limited overlap, thus partially preserving the cortical spatial organization (Figures 9, 10). Frontal cortex projects rostrally and medially in the PN, parietal cortex projects to central and caudal parts, temporal cortex to central and lateral regions and occipital cortex projects to lateral and rostral parts of the PN (Leergaard and Bjaalie, 2007). Although the projections are governed by a topographical pattern, convergent as well as divergent projections can be found (Mihailoff, 1983; Nikundiwe et al., 1994), the overall convergence of corticopontine to pontocerebellar neurons being 2:1 (Brodal and Bjaalie, 1992).

The topography of corticopontine projections is also maintained within the representation of the somatosensory modality. Tactile information from the body is somatotopically mapped onto the primary somatosensory cortex. This information is projected to the PN largely maintaining the somatotopy of the information (Leergaard et al., 2006). In support of this, pontine neuron dendritic fields remain largely constrained within the area targeted by a single cortical region (Schwarz and Thier, 1995). It is noteworthy, however, that some PN neurons have large dendritic fields, receiving information from various cortical locations, therefore suggesting that integration of sensory information might happen already at the level of the PN (Schwarz and Thier, 1995).

The concentric “inside-out” temporal gradient of neuronal organization in the PN might correlate with patterned axonal input related to temporal maturational gradients of layer V cortical neurons—referred to as the chrono-architectonic hypothesis (Altman and Bayer, 1997). The earliest arriving corticopontine fibers grow into the core of PN, where the earliest born PN neurons have settled. Step by step, the PN are innervated in an inside-out fashion. Consequently, a causal link between birthdate/arrival of PN neurons and birthdate/arrival of projections at the PN was proposed (Altman and Bayer, 1987d, 1997; Leergaard et al., 1995; Figures 9, 10). Also, cortical areas are broadly pre-patterned along the rostrocaudal axis by the graded activity of homeobox transcription factors (O’Leary et al., 2007). It is noteworthy that rostrocaudal pre-patterning and regionalization is also observed in the developing PN as described before, thus intersecting spatial dimension to the temporal gradient model (Figure 7). Both timing and rostrocaudal information could in turn generate a complex morphogenetic field that might contribute to the target selection of cortico-pontine and ponto-cerebellar fibers.

In contrast to most other precerebellar nuclei, PN also receive significant innervation from non-somatosensory cortices including the visual and auditory cortices—as shown in several mammalian species including rodents, rabbits, cats and monkeys. In particular, the RTN and the dorso-lateral pontine nucleus (DLPN) constitute a major relay for visual and visuomotor input into the cerebellum (Glickstein et al., 1980, 1994; Thielert and Thier, 1993). The DLPN has been particularly well analyzed. It receives major inputs from the visual cortex (Glickstein et al., 1980, 1994) and auditory cortex (Perales et al., 2006). The DLPN has been involved in optokinetic nystagmus including smooth-pursuit eye movements, ocular following, visually guided motor learning (Tziridis et al., 2009, 2012) and visually guided eye movements (May et al., 1988; Thier et al., 1988; Ono et al., 2003; Thier and Möck, 2006).

Several other subcortical and spinal cord regions are also known to provide input to the PN, however these projections account for only a small fraction of afferents to the PN. Subcortical structures projecting to the BPN have been broadly classified in two groups based on their projection pattern (Brodal and Bjaalie, 1992). The first group includes nuclei which project to BPN in a diffused manner. These nuclei include reticular formation, raphe nuclei, nucleus coeruleus and periaqueductal gray (summarized in Paxinos, 2014). The second group is composed of nuclei with at least partially topographic projections in the BPN. This group includes the superior colliculus, medial mammillary nucleus, trigeminal nucleus, dorsal column nuclei, the spinal cord, the pretectal nuclei, zona incerta and intracerebellar nuclei (Figure 10; summarized in Paxinos, 2014). It is interesting to note that in the BPN, specific subcortical afferents overlap with cortical afferents arising from regions which are functionally related. One of the best examples are projections from dorsal column nuclei that topographically overlap with the afferents from limb specific somatosensory cortex (Kosinski et al., 1986).

## Ponto-Cerebellar Projections of Pontine Nuclei

**Figure 11:**
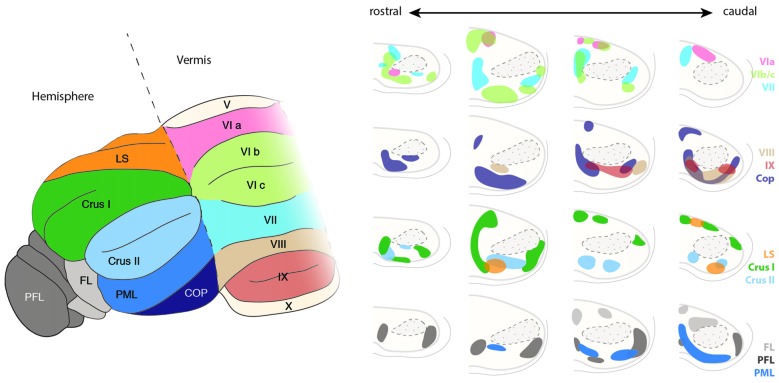
**Topography of the ponto-cerebellar projections**. The PN project to lobules VI to IX of the vermis as well as to the cerebellar hemispheres including LS, Crus I and II, PML, COP, PFL and FL. Projections from the rostral and lateral regions of the PN, corresponding to the visual areas, innervate mostly the PFL, whereas COP mostly receives projections from the rostral and dorsal region of the PN. The basal PN strongly innervate Crus I and II, PML as well as lobules VIb, c and VIII. In general, ponto-cerebellar projections display strong overlap suggesting distribution of the information from a single region in the PN to various lobules in the cerebellum. PN, pontine nuclei, LS, lobule simplex, PML, paramedian lobule, COP, copula, PFL, paraflocculus, FL, flocculus. (Adapted with permission from Paxinos, 2014, Elsevier).

While a broad topographic organization of the cortico-pontine projections is well established and described in detail (Leergaard et al., 2006; Leergaard and Bjaalie, 2007), the logic behind the connectivity patterns from PN to the cerebellum has been debated for many decades (Apps and Hawkes, 2009). Projections from PN to cerebellum have been intensively studied in rats (Azizi et al., 1981; Mihailoff et al., 1981) and monkeys (Brodal, 1982) by performing retrograde tracing experiments. BPN and RTN project to multiple locations in both cerebellar vermis and hemispheres (Azizi et al., 1981; Mihailoff et al., 1981; Paxinos, 2014; Figure 11). There is evidence for a high degree of divergence and convergence of projections/terminations of single PN neurons or clusters of PN neurons. Divergence is suggested by the fact that neurons located in close by regions within BPN or RTN are labeled by distant cerebellar injection sites, convergence is put forward by the fact that restricted cerebellar injections result in the retrograde labeling of spatially distinct neuron populations within BPN (Azizi et al., 1981; Mihailoff et al., 1981). A few single neurons can also project to multiple lobules (Bolstad et al., 2007). Therefore the complexity of the cerebrocerebellar connectivity seems to arise from properties of the PN that do not simply relay information from the cortex, but redistribute permutations of information from cortical sensory and motor input in a convergent and divergent manner to different areas of the cerebellum. Such connectivity patterns are essential for distributing information from discrete corticals regions to multiple lobules of the cerebellum. The cerebrocerebellar connectivity patterns have therefore both a partially topographic as well as a non-continuous or “fractured” organization.

PN mossy fibers have collateral branches targeting the deep cerebellar nuclei. Little overlap is observed between the termination zones of BPN and RTN neurons within cerebellar nuclei. BPN projects mostly to the lateroventral part of the nucleus lateralis and caudoventral part of the nucleus interpositus anterior. Projections from the RTN are mostly observed in the nucleus medialis and mediodorsal part of the nucleus lateralis (Cicirata et al., 2005). The number of cerebellar fibers arising from BPN are higher than those from RTN. The distinct connectivity of BPN and RTN cannot be explained on the basis of differences in the maturation of projection neurons in these nuclei as dendritic field, number of branching points, or the length of terminal dendrites of the neurons in BPN and RTN are similar (Schwarz et al., 1996). Although some differences were observed, such as larger somata and more primary dendrites in the projection neurons located dorsally than ventrally, these differences are more reflective of the dorsoventral positioning rather than nucleus-specific properties.

How the complex patterns of pontocerebellar connectivity are achieved is poorly understood. Most tracing data (Azizi et al., 1981; Mihailoff et al., 1981; Broch-Smith and Brodal, 1990) suggest that neither the temporal (inside-out) nor other spatial distribution of PN neurons might be major determinants for the establishment of the pontocerebellar connectivity patterns. Cells labeled by retrograde tracings of specific lobule do not cluster unambiguously along inside-out, mediolateral or rostrocaudal axes in the PN (Azizi et al., 1981; Mihailoff et al., 1981; Broch-Smith and Brodal, 1990). On the other hand, retrograde tracing experiments performed in cats (Broch-Smith and Brodal, 1990; Nikundiwe et al., 1994) have shown that more than 70% of the neurons labeled after injections in the paraflocculus are located in the rostral half of the PN and have a lamellar-like organization (Nikundiwe et al., 1994). The paraflocculus projecting areas of the PN mainly get inputs from visual, parietal associative and to a lesser extent from primary and secondary sensory and primary motor cortical areas, but almost no input from secondary motor areas and prefrontal cortex (Broch-Smith and Brodal, 1990; Nikundiwe et al., 1994). Interestingly, it has been also shown that the pattern of cerebropontocerebellar projections converge with somatotopically equivalent projections from the inferior olive (Odeh et al., 2005), suggesting common organizational principles across precerebellar nuclei.

## Evolution of The Pontine Nuclei and Cortico-Ponto-Cerebellar Connectivity

PN have been analyzed in mammals from rats (Mihailoff et al., 1978) to opossums (Mihailoff and King, 1975), cats (Hoddevik, 1975) and macaques (Glickstein et al., 1980). The PN are often considered as structures specific to mammals and have been poorly described in other amniotes such as reptiles and birds. If the structures found in amniotes are indeed PN homologous structures is debated. Two populations of PN, referred to as medial pontine nucleus and lateral pontine nucleus have been described in chicken (Brodal et al., 1950; Marín and Puelles, 1995). These two populations are located medially and laterally to the midline at the level of r3/r4 and originate at the posterior rhombic lip (Marín and Puelles, 1995). Birds neither have cortico-spinal tracts (Aboitiz et al., 2003) nor pronounced cerebellar hemispheres, their cerebella consisting almost entirely of vermis (Sultan and Glickstein, 2007). Therefore, PN neurons lack the main input (cortex) as well as their main output areas (cerebellar hemispheres). Nonetheless, they share some striking similarities with two nuclei that exist also in rodents, namely the RTN and the recently described interfascicular trigeminal nucleus (Fu et al., 2013). Both nuclei are strongly innervated by subcortical areas (and have only minor input from the cortex) and profusely project to the vermis in rodents (Azizi et al., 1981; Mihailoff et al., 1981; Fu et al., 2011). The mammalian RTN is for example involved in processing visual information and strongly innervated by subcortical afferents as e.g., from tectum (superior colliculus), by which it receives visual and oculomotor information (Thielert and Thier, 1993). Considering the importance of the visual and oculomotor system in amniotes in contrast to somatosensory and tactile information (Naumann et al., 2015), we speculate that a primitive mammalian ponto-cerebellar system might have processed only visual and vestibular information. Mammals as rodents and monkeys extensively use the somatosensory sense to experience their environment, especially by the use of whiskers (rodents and cats), lips and paws/hands (monkeys), which might explain the expansion of the cortico-ponto-cerebellar system and the stronger projections of somatosensory and motor areas.

Hence, the coevolution of cortex, PN and cerebellar hemispheres, the three components of the cortico-ponto-cerebellar circuit, may have played a pivotal role for the evolution of complex motor behaviors (including the corresponding sensory feedback), since all three areas increased dramatically in size throughout evolution. In humans, more than a third of the hindbrain is occupied by the PN, and both cortex and cerebellar hemispheres increased greatly in volume (the hemispheres are also referred to as cerebro- or neo-cerebellum). A further interesting observation on the evolution of the cortex is that the cortical area subdivisions well described in rodents, monkeys and humans (Rakic, 2009) are not as strictly topographically segregated in monotremes and marsupials, the sister groups of mammals (Lende, 1963, 1964; Krubitzer, 2007) and are absent in non-mammalian vertebrates (Aboitiz et al., 2003). Marsupials as the opossum and wallaby (Lende, 1963) and monotremes (Lende, 1964) have a strong to almost complete overlap of somatosensory and motor areas in the cortex (Aboitiz et al., 2003).

The spatial segregation of distinct somatic motor and sensory representations came along with the increasing size of cortical areas as well as with the interpretation of sensory information and the control of motor behavior (Aboitiz et al., 2003; Rakic, 2009). Interestingly, spatial segregation is less obvious in the cerebellum (Apps and Hawkes, 2009), due to the functional role of the cerebellum as a major integrator of motor and sensory information. The PN evolved during early mammalian evolution, at a time when neocortex and neocerebellum (the hemispheres) evolved from the dorsal pallium and in the cerebellum, respectively. Cortex and cerebellum have a strikingly correlated volumetric evolution (Barton, 2002) further suggesting a strong link between the evolution of these two brain regions. It is also likely that the main structure connecting these two areas has an important function during the evolution of these two systems. From an evolutionary-developmental perspective, it might be postulated that the highly convergent and divergent circuitry between cortex and cerebellum are reminiscent of the cortical features of evolutionarily early mammals in which somatosensory and motor areas were partially overlapping, as it is still in the PN of more derived mammalian species. Hence, it is possible that the evolution of an increased connectivity with the cerebellum resulted in a gradual transfer of integrative computations that were performed in the cortex of ancestral mammals to the cerebellum of derived mammals. From an evolutionary standpoint this could explain the gradual decrease of sensory topography along the cortico-ponto-cerebellar pathway. It coincides with stronger divergence and convergence of connections transforming the somatotopically continuous sensory maps in the cortex over PN to the fractured somatotopy in the cerebellum, serving to integrate sensory-motor information.

## Conclusions and Outstanding Questions

The PN are a suitable system to study processes of neuronal development from early specification to neuronal migration, axon guidance, target selection and synaptic refinement. Also, the PN can be considered as part of one of the most complex neuronal circuits within the brain—not only in terms of connectivity pattern but also function. Several questions remain that are of central importance for a better understanding of brain development and function. One of the most puzzling topics in developmental neuroscience is how complex circuitry emerges from homogenous cell populations. For the PN it has been suggested that both the rostrocaudal origin and the birthdate of PN neurons are determinants of their connectivity (Altman and Bayer, 1987d, 1997; Leergaard et al., 1995; Di Meglio et al., 2013). However, the molecular logic behind the complex input-output PN connectivity patterns has not been fully uncovered. For a better understanding of the molecular determinants of PN connectivity, a combination of tools and approaches will be necessary. First, approaches that combine connectivity pattern analysis with markers for birthdate, rostro-caudal origin and other available markers are necessary. Second, due to the complexity of the circuit, a focus on single PN neuron molecular identity and associated wiring will be required. Single-cell transcriptomics can help to understand the transcriptional diversity that in turn control differences in projection patterns and innervation among PN neurons. Also the wealth of transgenic lines that exist nowadays can be used in combination with *in utero* electroporation and transynaptic virus tracing (Wickersham et al., 2007) to analyze the connectivity and transcriptional profile of specific PN subsets. This includes rostrocaudal subsets that can be targeted using *Hox* gene enhancers driving Cre expression (Di Meglio et al., 2013), or PN neuron subsets with different birthdates that can be labeled using *in utero* electroporation at different embryonal stages or by *CreER*^T2^ lines (Machold and Fishell, 2005). Also the evolutionary origin of the PN and cortico-ponto-cerebellar connectivity would be an exciting research focus. With the expanding toolset for gene editing in non-model organisms this might become feasible (Kratochwil and Meyer, 2015).

These are exciting times in developmental neuroscience, where we can expect great advances in understanding the molecular determinants of complex neural circuitry. The PN are a wonderful system that allows studying all developmental processes from progenitor specification to synapse formation.

## Author Contributions

CFK, UM, and FMR conceived and discussed the topic, organization, and layout of the review article. CFK and UM wrote an initial draft of the manuscript and prepared the figures. FMR revised and finalized the article.

## Conflict of Interest Statement

The authors declare that the research was conducted in the absence of any commercial or financial relationships that could be construed as a potential conflict of interest.
